# Parosmia and Neurological Disorders: A Neglected Association

**DOI:** 10.3389/fneur.2020.543275

**Published:** 2020-11-09

**Authors:** Rosella Ciurleo, Simona De Salvo, Lilla Bonanno, Silvia Marino, Placido Bramanti, Fabrizia Caminiti

**Affiliations:** Istituto di Ricovero e Cura a Carattere Scientifico Centro Neurolesi “Bonino-Pulejo”, Messina, Italy

**Keywords:** parosmia, epilepsy, multiple sclerosis, traumatic brain injury, mesial temporal sclerosis (MTS), sniffin sticks test

## Abstract

Parosmia is a distorted olfactory sensation in the presence of an odor. This olfactory disorder can affect the quality of life of most patients who experience it. Qualitative olfactory dysfunctions, such as parosmia and phantosmia, may be clinical conditions secondary to neurological diseases. The incidence of parosmia is underestimated, as well as its association with neurological diseases, due to poor self-reporting of patients and lack of objective methods for its measure. In this paper, we show selected clinical cases of parosmia associated with neurological disorders, such as traumatic brain injury and multiple sclerosis. These clinical cases show how the correct diagnosis of parosmia can represent the tip of the iceberg of important underlying neurological disorders and be a good prognostic indicator of their progression or recovery.

## Introduction

The smell, together with the sight and hearing, is one of the special senses used to monitor the human environment and to provide an escape route from dangerous situations (1).

Although the sense of smell has always been considered with less interest than the other senses, it plays a pivotal role in ordinary life. Indeed, the loss of smell or its alteration can affect the quality of life (QoL) significantly (2).

**Figure 1:**
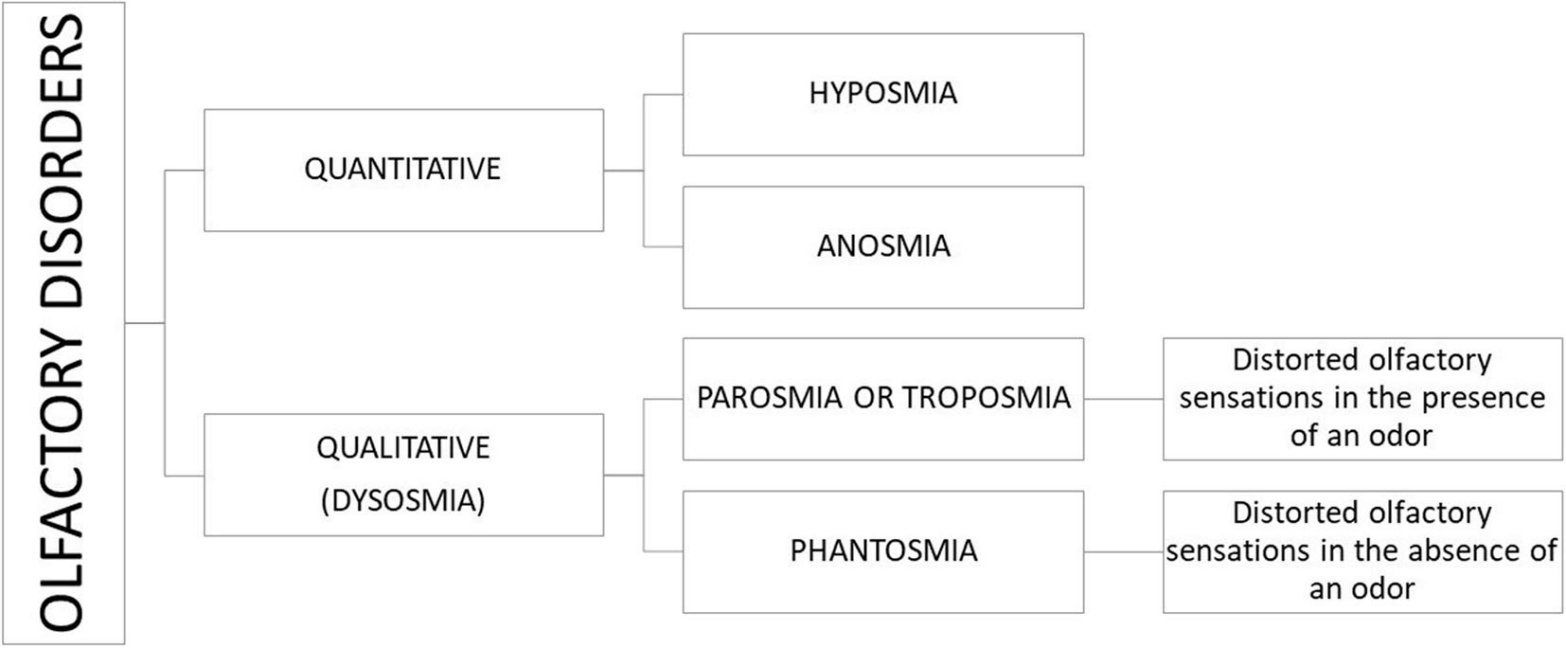
Classification of olfaction disorders.

The disorders of the olfaction can be classified as quantitative (hyposmia and anosmia) and qualitative dysfunction. The qualitative olfactory dysfunction (dysosmia or olfactory distortion) can be divided into parosmia and phantosmia (3) (Figure 1).

Hyposmia is a partial loss of smell, whereas anosmia is the total inability to perceive the odorants. Parosmia is a distorted smell perception in the presence of an odorant stimulus. Phantosmia is an olfactory hallucination perceived when no odorants are present. Both the olfactory distortions are typically described as unpleasant (rotten, sewage, or burn smell) (4).

It has been widely reported that parosmia may significantly worsen the QoL because of foul odor and altered taste that can lead to avoid some foods and then to weight loss (5). Indeed, taste alterations often reflect olfactory dysfunctions (6). Moreover, some authors have found that the patient's discomfort due to the olfactory distortion weighs more than the isolated olfactory loss (7).

To date, the lack of specific olfactory tests for the evaluation of the parosmia and its poor self-reporting do not allow a real estimate of its incidence. In clinical practice, it occurs rather frequently that the patient reports a distortion of smell and literature data support a prevalence of the parosmia in the following conditions: head trauma (29–55%), post-upper respiratory tract infection (35–51%), sinonasal diseases (17–28%), toxins/drugs (17–28%) (8), and temporal lobe epilepsy (0.6–16%) (9). If, on the one hand, the incidence of parosmia is underestimated, on the other hand, the estimate of parosmia association with neurological pathologies is even more so.

In this paper, we show some selected clinical cases of parosmia associated with neurological diseases, underlining the value of the clinical evaluation, in terms of anamnesis, examinations, and testing, for its correct diagnosis.

## Parosmia and Neurological Disorders

Parosmia may be a secondary disorder to neurological diseases. Several studies evaluated the possible association of the parosmia with epilepsy, traumatic brain injury (TBI), and multiple sclerosis (MS).

### Epilepsy

The temporal lobe plays an important role in the olfactory system. In particular, the entorhinal cortex, piriform cortex, and amygdala are involved in the perception (10), identification, discrimination of odors, and consolidation of the olfactory memory (11). Several studies have shown that the temporal mesial structures (especially the amygdala) are crucial in the genesis of olfactory auras, either as primary focus or as relay station for the diffusion of seizure discharges (9). In line with these findings, Jacek et al. (12) described the case of a man affected by temporal lobe epilepsy with an ictus-related parosmia. If, on the one hand, the patient did not exhibit any abnormalities in odor identification and only few evidences of decreased discrimination of familiar odors, on the other hand, he reported altered perception of odor quality and hedonics provoking nausea and gagging, probably due to a strong change in his limbic response to smells. This finding could suggest the major role of the amygdala in the hedonic processing of unpleasant odors (13).

Brief olfactory auras before the beginning of seizure (14) or chronic olfactory disorders have been widely described, especially in odor identification and discrimination domains (12, 15, 16). It is worthy to note that the olfactory auras are often accompanied by other kinds of aura (psychiatric, autonomic, visual, and gustatory auras) (11). Temporal mesial epilepsy (TSE) studies have proven that there was no difference in olfactory alterations between the right and left TSE, supporting the hypothesis that the olfactory processes involve the activation of both hemispheres (17). Our clinical case of parosmia (16) found an abnormal reduction of left hippocampus volume associated with the unilateral deficit of identification only in the side ipsilateral to imaging findings. In addition, on suspicion it was a case of mesial temporal sclerosis (MTS), the therapy with levetiracetam for 6 months improved the parosmia symptom and increased the score of identification test, supporting the central genesis of parosmia and its role of early symptom in this neurological disorder.

It has been demonstrated that unilateral temporal lobectomy in epilepsy patients has led to significant worsening of bilateral olfactory function with lower olfactory scores on the resection side compared with the non-lesioned side (10). These findings are in agreement with previous evidences, supporting the dominant role of the right temporal lobe in olfactory function (18, 19). Conversely, lesion and functional brain imaging studies of healthy subjects suggested that olfactory function requires input from both the left and right temporal lobe regions for optimal odor recognition, thus ruling out a strong functional lateralization for olfactory memory function (20).

### Traumatic Brain Injury

TBI is one of the most common causes of olfactory dysfunction, including both quantitative and qualitative smell disorders.

Post-traumatic olfactory impairment can be due to several factors, ranging from damage of the olfactory neuroepithelium and/or of olfactory nerve filaments up to lesions, hurts, and bleeding in olfactory-related brain structures, such as the orbitofrontal cortex and the medial temporal lobe (21–23).

Although TBI usually results in a quantitative smell loss, quantitative and qualitative olfactory disturbances are often found in combination (4). The temporal relationship between parosmia and quantitative olfactory dysfunction is not simple and still a matter of debate. Indeed, it has been previously reported that the parosmia may occur simultaneously or after smell loss, but not before (4). Moreover, Doty et al. (24) found that the prevalence of patients experiencing parosmia reduces from 41.1 to 15.4% over an 8-year post-trauma period, thus suggesting that post-traumatic parosmia ameliorates over time.

Parosmia in patients with TBI can have a genesis related to central and peripheral damage. The peripheral lesions, due to trauma to the face and brain, may reflect an incomplete representation of the odor, whereas central lesions may cause chaotic processing of odors at the higher levels (3, 7, 25). A magnetic resonance imaging (MRI) study in patients with post-traumatic olfactory loss showed that olfactory bulb volume was smaller in patients with parosmia than in those without, and that the presence of parosmia was clearly associated with the presence of cerebral damage, especially in the orbitofrontal and anterior temporal cortices (26). Another neuroimaging study in patients with TBI-related olfactory dysfunction reported that post-traumatic parosmia required at the same time the presence of damaged brain regions, such as the medial or lateral orbitofrontal cortices, and intact others, such as the lateral orbitofrontal cortex and temporal lobe pole (27). The detection of a partial intact olfactory system supports the hypothesis of compromised interplay among its central components.

### Multiple Sclerosis

To our knowledge, few cases of parosmia associated with MS were reported in the literature. A case report described a 36-year-old woman who developed parosmia 3 months before MS diagnosis. She perceived the food as smelling of gasoline. Consequently, she developed an aversion to food and lost 9 kg. The treatment with methylprednisolone improved her MS and olfactory symptoms (28). A study that evaluated the ortho- and retronasal olfactory functions in MS patients found that 75% of the 16 investigated patients showed a quantitative olfactory disorder, 6.25% reported parosmia, and 18.75% reported phantosmia (29).

### Parkinson's Disease

Olfactory dysfunctions are often associated with Parkinson's disease (PD). The prevalence of olfactory dysfunctions in PD ranges from 45 up to 90%, representing one of the top five most prevalent motor and non-motor symptoms affecting the QoL of patients (30). In the early phases of the disease, the quantitative olfactory dysfunction precedes the onset of motor symptoms (31). Along with hyposmia or anosmia, phantosmia (32–34), but not parosmia, has also been suggested to herald PD. Indeed, a study on 474 patients diagnosed with PD 15 years after the finding of idiopathic smell disorder reported that PD development was associated with anosmia and hyposmia, but not to qualitative smell loss (35).

## Clinical Evaluation of Parosmia

In the absence of a standardized test for the parosmia, the diagnosis is based on parosmic experience reported by the patient. Instead, a qualitative method to evaluate the olfactory function is the Sniffin' Sticks Test (SST) (36–39). In particular, the sub-test of identification could indicate if the recognized odors are different compared with those inhaled. Unfortunately, an objective measure of qualitative olfactory distortion is so far not available.

**Figure 2:**
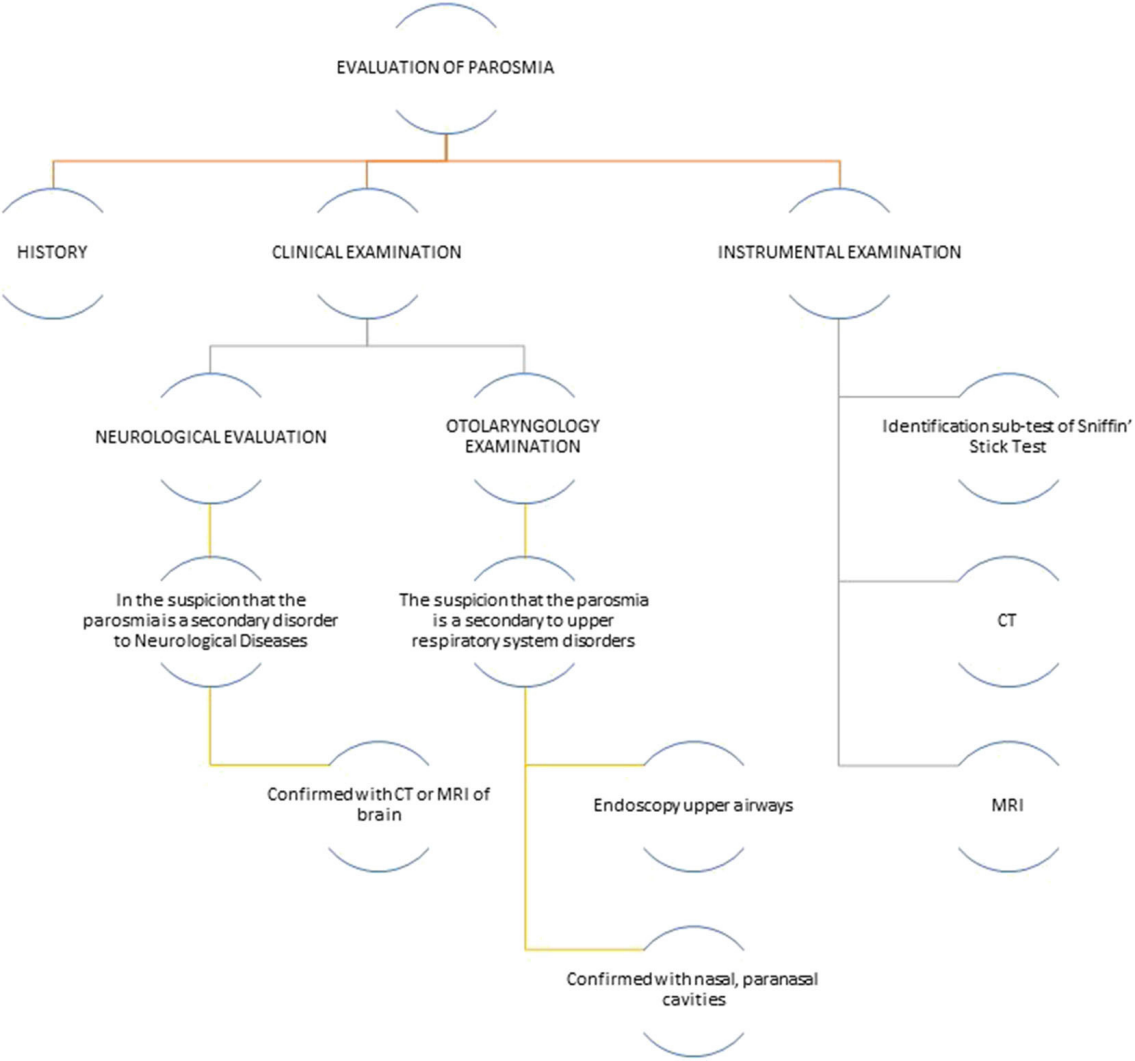
Flowchart of olfactory disorders assessment. CT, computed tomography; MRI, magnetic resonance imaging.

To evaluate if the parosmia is a secondary disorder to neurological diseases, it is needed to perform an assessment based on history, clinical and instrumental neurologic examination, and smell testing (Figure 2). The medical history should collect patient's self-reporting, the presence of current or recurrent allergic rhinitis and sinus disorders, any recent or past head trauma, smoking history, alcohol abuse, concomitant medications, any weight loss, and/or change in food habits. In addition, an evaluation on memory and cognitive performance would be advisable. A clinical and instrumental otolaryngology evaluation is needed to exclude that the cause of parosmia is upper respiratory tract infection or sinonasal diseases. For patients with confirmed head trauma, epilepsy, or MS, brain MRI could be useful in order to localize a possible lesion of the olfactory system and, then, postulate that parosmia is caused by it.

## Case Report

### Patient 1

A 40-year-old woman presented with a complete olfactory loss 6 months after a head trauma due to a car accident. Her olfactory function was studied by using the SST that showed a final threshold, discrimination, and identification (TDI) score of “10,” confirming the diagnosis of bilateral anosmia. A MRI examination confirmed the presence of a post-traumatic scarring localized in the right orbitofrontal region.

During the 6-month follow-up period, the patient's quantitative olfactory function improved, as demonstrated by the SST score (final TDI score = 24), confirming a diagnosis of functional bilateral hyposmia. However, at the time of olfactory loss recovery, the patient recognized an unpleasant odor of sulfur whenever she smelled perfumes. This case reports a clear association between the quantitative olfactory recovery and the onset of post-traumatic parosmia.

### Patient 2

A 35-year-old man reported a complete loss of smell associated with parosmia 1 month after he was run over by a car while crossing a road. This incident caused him a TBI. In particular, he recognized a bad odor of rotten food whenever he smelled some foods, such as meat. So, he initiated a vegetarian diet. The SST showed a final TDI score of “17,” confirming a diagnosis of severe bilateral hyposmia. The upper airway fibroscopy and the CT scan of the nasal and paranasal cavities and of the skull base have excluded infections or other causes of olfactory dysfunction. A brain MRI revealed a little scarring localized in the left temporal and in the right frontal regions. In the follow-up visits, 6 months and 1 year after the first assessment, the patient did not report any improvement of hyposmia. However, he reported a gradual decrease of parosmia severity following the instilling of salty water in the nasal cavities. This clinical result could support both central and peripheral genesis of the parosmia after head trauma.

### Patient 3

A 22-year-old female patient developed chronic parosmia 1 year before our evaluation. She reported perceiving an olfactory sensation difficult to explain when she smelled food. For this reason, she lost 8 kg of her body weight. The SST showed a final TDI score of “28,” confirming a mild bilateral hyposmia. The absence of otorhinolaryngological diseases, no head trauma history, or other causes of olfactory disorders have made necessary an in-depth brain MRI that detected multiple lesions in the white matter, compatible with MS. Later, a neurological examination confirmed the diagnosis of MS. This is a rare case in which the parosmia is an earlier manifestation of MS.

### Patient 4

A 36-year-old male patient affected by MS, clinically stable (Expanded Disability Status Scale = 1.0), was referred to our clinic for parosmia. In the recent months, he perceived bad smells during cooking, which increased in duration and intensity. The upper airway fibroscopy and the CT scan of the nasal and paranasal cavities and of the skull base excluded infections or other causes of olfactory dysfunctions. We excluded that the parosmia was related to exposure to toxins, head trauma, and therapy for MS (interferon beta) that he started 5 years earlier when MS was diagnosed. The scores obtained by administering SST in birhinal mode were as follows: for threshold sub-test “8,” for discrimination sub-test “9,” and for identification sub-test “11,” with a final TDI score of “28,” which confirmed a mild functional bilateral hyposmia. During the olfactory test, 5 (garlic, fish, coffee, turpentine, and mint) of 16 odorants in the identification sub-test were perceived by the patient as other bad odors. Assuming that the patient's parosmia could be ascribed to MS, he underwent a brain MRI that revealed the appearance of new lesions in the orbitofrontal cortex. Corticosteroid therapy for 7 days improved the parosmia, indicating a good correlation between MS radiological relapse and olfactory dysfunction.

## Discussion

Olfaction is a physiologic process that provides important information about the surrounding environment. Odor perception starts when the odorant molecules stimulate the primary olfactory receptors contained in the neuroepithelium localized in the superior nasal cavity adjacent to the cribriform plate, superior nasal septum, and superior-lateral nasal wall. Odorants can also be perceived *via* the back of the throat when food and liquids are consumed (retronasal olfaction). The olfactory cells pass their signals to the olfactory bulb through the cribriform plate of the ethmoid bone. In the olfactory bulb, the olfactory information is transducted and coded before it is processed and disseminated to other areas of the central nervous system. Cortical regions receiving projections from the olfactory bulb include those collectively termed the “primary olfactory cortex,” namely, the anterior olfactory nucleus, tenia tecta, olfactory tubercle, piriform cortex, anterior cortical amygdaloid nucleus, periamygdaloid and entorhinal cortices, and amygdala.

**Figure 3:**
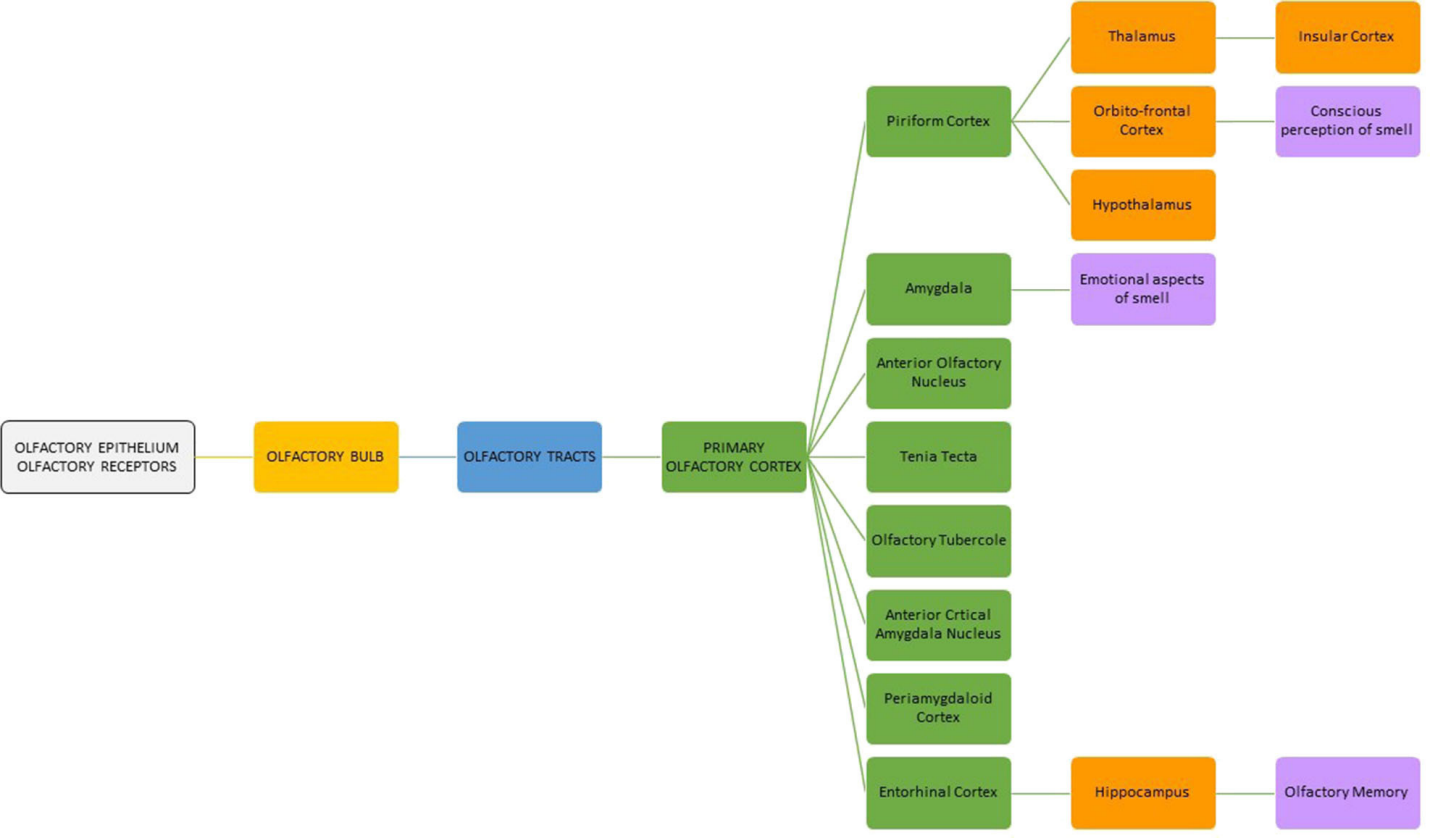
Flowchart of olfactory pathway.

The piriform cortex is connected to the “secondary olfactory areas,” such as the thalamus, hypothalamus, and orbitofrontal cortex. The nuclei of the thalamus have further connections toward the orbitofrontal and insular cortices. From the entorhinal cortex, fibers lead to the hippocampus. The primary and secondary areas may play a role in olfactory pleasure sensation, memory, emotional response to odors, and mood (Figure 3) (40, 41).

The diagnosis of qualitative olfactory dysfunctions needs to be carried out by the clinician in a careful, objective and systematic manner, since they might signal severe diseases. In particular, the parosmia may be a secondary disorder to neurological diseases or even an only early manifestation. Indeed, in clinical cases of MTS (16) and MS, parosmia has been shown to be a prodromal symptom of these neurological disorders, supporting the pivotal role of neurological assessment in cases of qualitative olfactory disorders where no other systemic diseases are associated.

Interestingly, the parosmia may be associated more often with a quantitative olfactory loss (4), though it may occur among subjects with normal olfactory function (42, 43). It seems that the parosmia occurs during neuronal death when it is associated with a quantitative olfactory loss (7). In our cases, the parosmia was always associated with functional olfactory loss. However, in the first case of head trauma, the parosmia onset was concomitant with the recovery of olfactory loss. This recovery could be ascribed to a regeneration of new cells of the olfactory neuroepithelium that, for unknown reason, they do not encode the odorant as before. Therefore, the parosmia could be an expression of olfactory system change (regeneration) or as far as of a recovery sign. Although it seems that parosmia can be regarded as an indicator of early recovery of quantitative olfactory dysfunction, a study by Reden et al. (25) indicated that the occurrence of parosmia has little prognostic value.

The underlying pathophysiological mechanisms of the parosmia are still a matter of debate. The partial loss of olfactory receptor neurons, the interneuronal loss responsible for dysfunctions of the olfactory bulb, abnormalities in axonal targeting from regenerating fibers after injury, or the pathology of the interpretive central nervous system could be based on the pathophysiological mechanisms of the parosmia (44). These mechanisms could explain the association of parosmia with quantitative olfactory disorders. However, if, on the one hand, the “peripheral theory” proposes the inability of abnormal cells of the olfactory neuroepithelium to determine a complete characterization of the odorant, on the other hand, the “central theory” suggests an alteration of cerebral integrative centers of the smell (42). The “peripheral theory” is supported by the association of parosmia to olfactory neuroepithelium changes after upper respiratory tract infection or head trauma (45).

Even the elements supporting the central role of parosmia are few. Among these, there is the smaller olfactory bulb volume found in hyposmic patients with parosmia compared with hyposmic patients without parosmia (46). It has been hypothesized that the interneuron decrease in the olfactory bulb might result in a decrease of neuronal inhibition that in turn might be responsible for an aberrant olfactory partner. Parosmia may lead to specific changes in the primary and secondary olfactory areas also. In MRI study (47), the whole brain analysis found gray matter volume loss in the left anterior insula of parosmic patients compared with healthy controls. In addition, a further analysis of volume of interest found volume loss in the right anterior insula, the anterior cingulate cortex, the hippocampus bilaterally, and the left medial orbitofrontal cortex. Because many of these areas are critically involved in olfactory quality discrimination and odor memory, it has been speculated that the parosmia was associated with structural changes in a neuronal network crucial for odor discrimination and memory. A functional MRI study demonstrated that hyposmic patients with parosmia exhibited a strong activation in the thalamus, known to be related to directed attention in relation to the sense of smell, and the putamen that is implicated in the recognition of disgust signals (48).

Our clinical cases have highlighted the heterogeneous presentation of the parosmia when it is secondary to neurological disorders. Although the parosmia is often underestimated by both the patient and the clinician, its careful evaluation, based on anamnesis, otolaryngological and/or neurological evaluation, imaging, and olfactory tests, may reveal other medical challenges and, at the same time, may lead to a correct diagnosis of the neurological diseases that cause the parosmia, especially when it is their early manifestation. In addition, the evaluation of the parosmia can be a good prognostic indicator of progression or recovery of some important neurological diseases.

## Data Availability Statement

No datasets were generated for this study.

## Ethics Statement

Written informed consent was obtained from the individuals for the publication of any potentially identifiable images or data included in this article.

## Author Contributions

RC: contributions to the conception or design of the work and wrote the manuscript. SD: the acquisition of data for clinical cases. LB: wrote sections of the manuscript. SM and PB: manuscript revision. FC: contributions to the conception or design of the work, the acquisition of data for clinical cases, wrote the first draft of the manuscript, and manuscript revision. All authors contributed to the article and approved the submitted version.

## Conflict of Interest

The authors declare that the research was conducted in the absence of any commercial or financial relationships that could be construed as a potential conflict of interest.
